# Proteomic Biomarkers Panel: New Insights in Chronic Kidney Disease

**DOI:** 10.1155/2016/3185232

**Published:** 2016-09-07

**Authors:** Simona Mihai, Elena Codrici, Ionela Daniela Popescu, Ana-Maria Enciu, Elena Rusu, Diana Zilisteanu, Radu Albulescu, Gabriela Anton, Cristiana Tanase

**Affiliations:** ^1^Victor Babes National Institute of Pathology, Biochemistry-Proteomics Department, Splaiul Independentei 99-101, Sector 5, 050096 Bucharest, Romania; ^2^Cellular and Molecular Medicine Department, Carol Davila University of Medicine and Pharmacy, No. 8 B-dul Eroilor Sanitari, Sector 5, 050474 Bucharest, Romania; ^3^Fundeni Clinic of Nephrology, Carol Davila University of Medicine and Pharmacy, Șoseaua Fundeni 258, Sector 2, 022328 Bucharest, Romania; ^4^Fundeni Clinical Institute, Nephrology Department, Șoseaua Fundeni 258, Sector 2, 022328 Bucharest, Romania; ^5^National Institute for Chemical Pharmaceutical R&D, Pharmaceutical Biotechnology Department, Calea Vitan 112, Sector 3, 031299 Bucharest, Romania; ^6^Stefan S. Nicolau Institute of Virology, Molecular Virology Department, Șoseaua Mihai Bravu 285, Sector 3, 030304 Bucharest, Romania; ^7^Faculty of Medicine, Titu Maiorescu University, Strada Dâmbovnicului 22, Sector 4, 040441 Bucharest, Romania

## Abstract

Chronic kidney disease, despite being a “silent epidemic” disease, represents one of the main causes of mortality in general population, along with cardiovascular disease, which is the leading cause of poor prognosis for these patients. The specific objective of our study was to characterize the relationship between the inflammatory status, the bone disorders markers, and kidney failure in chronic kidney disease patient stages 2–4, in order to design a novel biomarker panel that improves early disease diagnosis and therapeutic response, thus being further integrated into clinical applications. A panel of proteomic biomarkers, assessed by xMAP array, which includes mediators of inflammation (IL-6, TNF-*α*) and mineral and bone disorder biomarkers (OPG, OPN, OCN, FGF-23, and Fetuin-A), was found to be more relevant than a single biomarker to detect early CKD stages. The association between inflammatory cytokines and bone disorders markers, IL-6, TNF-*α*, OPN, OPG, and FGF-23, reflects the severity of vascular changes in CKD and predicts disease progression. Proteomic xMAP analyses shed light on a new approach to clinical evaluation for CKD staging and prognosis.

## 1. Introduction

Nowadays, chronic kidney disease (CKD) represents a worldwide major public burden and its prevalence continues to rise [1].

Over the past century, CKD, despite being a “silent epidemic” disease, represents one of the main causes of mortality in general population, alongside neoplasia, cardiovascular diseases, malnutrition, and infection, in the context of epidemiology landscape. Moreover, in Europe, CKD stages 1–5 prevalence ranges from 3.3% to 17.3% [2, 3].

Cardiovascular disease remains one of the leading causes of CKD poor prognosis, since early stages of CKD are associated with higher risk of subsequent coronary heart disease [4].

According to several clinical studies, 50% of patients with CKD die of cardiovascular causes, such as advanced calcific arterial and valvular disease; nonetheless, the processes of accelerated calcification in CKD remain poorly understood, and no therapies have been developed yet for disease prevention [5].

In CKD patients, screening for the presence of vascular calcification (VC) is suggested in current guidelines, since it is considered to be a cardiovascular risk marker and it is associated with a severalfold increase in morbidity and mortality risk, both in general population and in CKD, increasing steadily through the stages of CKD, peaking in CKD stage 5 patients.

Several proteins and factors are involved in passive and active processes that result in VC. In CKD population, various studies have identified circulating biomarkers that may be responsible for extraskeletal calcification and dysfunctions in mineral metabolism, which are features of CKD-mineral bone disorder (CKD-MBD) [6, 7].

Therefore, these observations have led to CKD-MBD study in association with cardiovascular diseases. These processes are interconnected and they have an important contribution to the morbidity and mortality rate of CKD patients [8, 9].

One of the main objectives in CKD therapy should be treating renal bone disease. The evaluation of CKD-MBD biochemical parameters (primarily phosphorus, calcium, parathyroid hormone, and vitamin D levels) as early as CKD stage 3, along with the assessment of bone status, should be considered in treatment decisions [10]. The prevalence of VC increases throughout the stages of CKD peaking in CKD stage 5 patients [7].

Cardiovascular calcification is an outstanding element of chronic inflammatory disorders associated with significant morbidity and mortality. Remarkably, CKD hastens atherosclerosis development and it has been demonstrated that CKD provokes excessive vascular inflammation and calcification [11].

Recent evidence also points towards alternative processes independent of osteogenic differentiation, including the release of matrix vesicles (e.g., secreted by macrophages) [5]. The understanding of the relationships between these mechanisms and signaling pathways could offer new mechanistic insight into the calcification process, and it may help lead to cardiovascular disease therapeutics in CKD patients [5, 11].

These data are also supported by genetic predisposition. Rutsch et al. [12] observed that 40–50% of coronary calcification cases can be attributed to genetics and several loci linked to coronary arterial calcification were identified [13, 14]. An implication of several single polymorphisms located at 9p21 locus near the cyclin genes has been suggested in the genesis of this pathology. These genes encode cyclins that may be broadly linked to cellular senescence and inflammation, though the accurate causative DNA sequences remain debatable [14, 15].

CKD is characterized by progressive loss of renal function, which results in reduced glomerular filtration rate (eGFR). Current clinical methods are accurate in diagnosing only advanced kidney dysfunction. In addition, there are no tools for predicting progression risk towards end-stage renal failure; thus, developing accurate biomarkers for prognosis of CKD progression constitutes a clinical challenge. Therefore, efforts are directed towards earlier detection and better prognosis, in order to allow better therapeutic interventions to slow down or even prevent the progression of the disease in the future. Omics approaches, including proteomics, provide novel insights into disease mechanisms. They may improve CKD management, providing stage-specific biomarkers [16–19].

In comparison with currently available markers, serum creatinine and urinary albumin, proteomic biomarkers may enable more accurate and earlier detection of renal pathology. Despite the “breaking point” being different in every patient, in some individuals serum creatinine levels remain normal despite loss of >50% of renal function; consequently, additional biomarkers of renal function are needed. Biomarkers that would facilitate the noninvasive differential diagnosis of kidney diseases, detect early onset of kidney disease, monitor responses to therapy, and predict progression to hard end points, such as end-stage renal disease (ESRD) or death [7, 20, 21], are needed, since they have potential for actual clinical implementation, which is an area to focus research on in the future [20, 22–25]. These biomarkers could prove very useful in terms of early detection and prognosis in CKD [7, 20, 26–31].

Considering the above-mentioned aspects, the specific objective of this study was to characterize the relationship between the inflammatory status and the indicators of kidney failure and bone disorders, in order to design a novel biomarkers panel that might improve early disease diagnosis and therapeutic response, thus being further integrated into clinical practice.

## 2. Materials and Methods

### 2.1. Patients and Samples

#### 2.1.1. Study Population

We prospectively included 86 patients (28% female and 72% male; mean age 65) diagnosed with chronic kidney disease according to the KDIGO criteria, 20 with CKD stage 4 (35% female and 65% male; mean age 62), 52 with CKD stage 3 (33% female and 67% male; mean age 66), and 14 with CKD stage 2 (23% female and 77% male; mean age 65), hospitalized in Fundeni Clinic of Nephrology, Fundeni Clinical Institute, Bucharest, and 20 healthy controls. Before enrollment, written informed consent was obtained from all subjects, according to Helsinki Declaration and Ethics Committee that has approved the study. Patients with acute infection, known malignancy, acute heart failure, significant heart valvular disease, and chronic use of glucocorticoids and immunosuppressive agents were excluded.

#### 2.1.2. Clinical and Laboratory Assessment

Clinical and anthropometric data were collected on the day of blood sampling: age, sex, weight, height, previous medical history, and concomitant treatment. Laboratory tests included hemoglobin, hematocrit, serum creatinine, urea, uric acid, glucose, total cholesterol, triglycerides, alkaline phosphatase, phosphate, calcium, albumin, and fibrinogen. Estimated glomerular filtrate rate (eGFR) was calculated using CKD-EPI formula. Urinary protein excretion was measured from a 24 h urine sample. All blood samples were collected in the morning after an overnight fast and were stored at −80°C until being analyzed.

### 2.2. xMAP Array and ELISA Analysis

The xMAP array was performed according to the manufacturers' protocols, and the plates were analyzed using Luminex 200 system. Cytokine levels and bone metabolism analytes were determined using the Milliplex MAP Human Bone Magnetic Bead Panel Kit from Merck-Millipore, Billerica, MA, USA, with 6 analyte-specific bead sets (simultaneous quantification): proinflammatory cytokines IL-6 and TNF-*α* and bone metabolism and disorder biomarkers: Osteoprotegerin (OPG), Osteocalcin (OCN), Osteopontin (OPN), and Fibroblast Growth Factor 23 (FGF-23). Briefly, the beads, which were provided within each kit, were incubated with buffer, cytokine standards (included in the kit), or samples in a 96-well plate at 4°C overnight. All further incubations with detection antibodies and Streptavidin Phycoerythrin Conjugate (SAPE) were performed at room temperature in the dark with shaking at 800 rpm. Multiplex data acquisition and analysis were performed using xPONENT 3.1 software; the calibration curves were generated with a 5-parameter logistic fit.

Fetuin-A serum levels were assessed using Quantikine® ELISA Human Fetuin A Immunoassay kit, R&D Systems, Inc., USA, according to the manufacturer's instructions.

Duplicate samples were used for all specimens and the average concentrations were used for statistical analysis.

### 2.3. Statistical Analysis

Differences between CKD sample group and control were analyzed using Student's *t*-test. A two-tailed *p* < 0.05 was considered statistically significant. The chi-square test (*χ* ^2^; *P*) was used to determine the significance of the association between inflammatory cytokines, bone metabolism, and disorder biomarkers in CKD and control groups. Pearson correlation (*r*) was used to explore the association between different biomarkers expression, together with clinical parameters. The threshold values for the analyses were established in accordance with the mean values of the studied groups. Statistical analysis was performed using SPSS 19.0 software. Graphs were realized with GraphPad Prism software (GraphPad Software Inc., La Jolla, CA).

## 3. Results and Discussion

Given the fact that a large number of cytokines orchestrate the inflammatory response, the extent to which inflammation plays a role in increasing the risk of bone/mineral disorders in CKD remains unclear. Progressive renal failure in CKD contributes to abnormalities in mineral/bone metabolism—calcium, phosphorous, PTH, Vitamin D, and vascular calcifications [32].

### 3.1. Inflammation and CKD

Inflammation represents a hallmark of CKD and the degree to which inflammation is related to loss in kidney function, eGFR, remains an open question. Some studies revealed increased circulating levels of proinflammatory cytokines IL-6 and TNF-*α* in patients with kidney dysfunction [33]. Moreover, inflammation status in CKD seems to be correlated with CKD evolution and complications, like cardiovascular disease [33, 34].

Okada et al. study supported the assumption that IL-6 genetic variations may lead to CKD and the assessment of the genotypes involved could identify the risk of CKD development [35]. Barreto et al. also showed that IL-6 levels tend to rise as CKD progressed, with the increase becoming statistically significant in CKD stages 4 and 3 [36].

**Figure 1:**
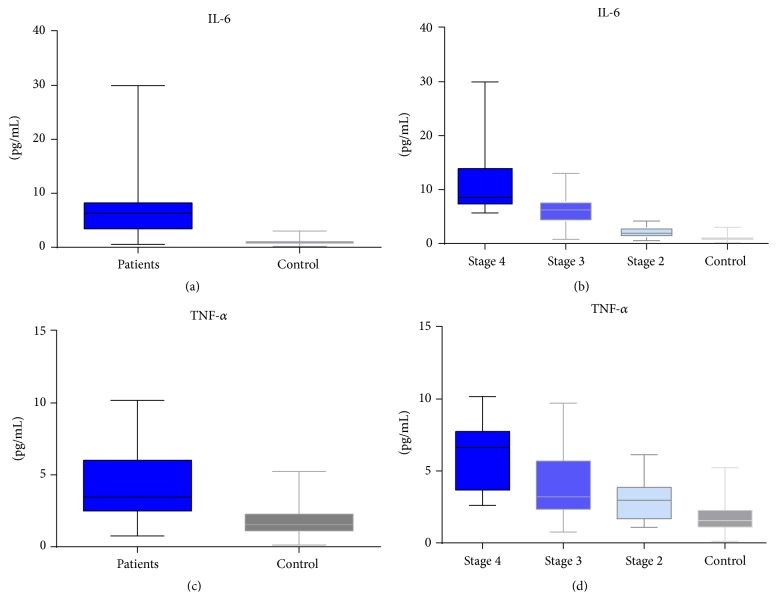
Serum levels of proinflammatory cytokines IL-6 and TNF-*α*, in CKD patients* versus* control, assessed by xMAP array.

**Figure 2:**
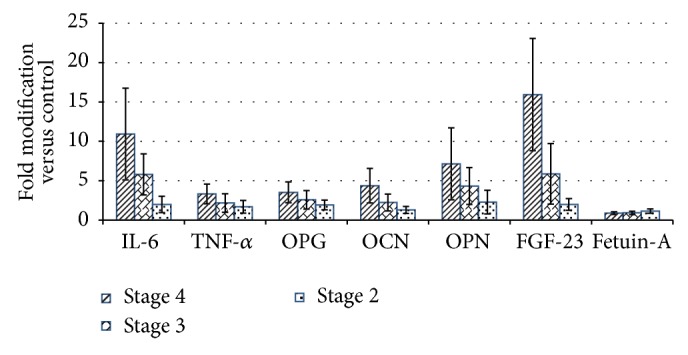
Modulation of serum biomarkers level in CKD stages. The data represent group averages of fold modification* versus* controls with standard deviations.

According to our data in this study, the mediators of the inflammatory response IL-6 and TNF-*α* have been overexpressed in all CKD groups (*t*-test; *p* < 0.001; Figures 1(a) and 1(c)). Statistical analysis (*t*-test) shows that IL-6 level was highest in CKD stage 4 (*p* < 0.001), being 11-fold higher than control, while, for CKD stages 3 and 2, the expression was also increased, 6-fold (*p* < 0.001) and 2-fold (*p* = 0.005), respectively, by comparison with control group (Figures 1(b) and 2).

In CKD stage 4, IL-6 enhanced expression was directly correlated with TNF-*α* (*r* = 0.64), OCN (*r* = 0.67), and OPN levels (*r* = 0.59) (Pearson correlation). In stage 2 of CKD, we have noticed a positive correlation between IL-6 and TNF-*α* (*r* = 0.58), OPG (*r* = 0.63), and OPN (*r* = 0.52), and, on the other hand, a negative correlation with Fetuin-A (*r* = −0.5) (Pearson correlation), as it is depicted in Tables 1, 2, and 3.

TNF-*α* displays an increased level in serum of more than 3.6-fold (*p* < 0.001) in CKD stage 4, recording a decrease with disease stage decrease as well, as follows: 2.8-fold (*p* = 0.006) in CKD stage 3 and 1.7-fold in CKD stage 2 (*p* = 0.01); these results are also related to renal failure (eGFR). Details on expression of TNF-*α* are provided in Figures 1(d) and 2.

In CKD stage 4, TNF-*α* enhanced expression is positively correlated with OCN (*r* = 0.69) and with OPN (*r* = 0.72) and negatively correlated with Fetuin-A (*r* = −0.62). In CKD stage 2, TNF-*α* was positively correlated with OPN (*r* = 0.51), as it is shown in Tables 1, 2, and 3.

It has been shown that IL-6, a “bad” cytokine that could promote atherosclerosis [37], might be more helpful than TNF-*α* in CKD patients classification on stages [38].

Spoto et al. also concluded that inflammation is related to renal failure, with high IL-6 levels seen in CKD early stages exclusively; however, their data showed a negative correlation between TNF-*α* levels and eGFR, indicating differences in the dynamics of the relationship between the above-mentioned cytokines and renal function [39]. Our data is in accordance with the increase of IL-6 in CKD, but we found a positive correlation between TNF-*α* and renal function.

The increased serum levels of IL-6 and TNF-*α* in CKD are in accordance with other studies, which mention that proinflammatory cytokines increase is linked to disease progression [40].

### 3.2. Markers of Mineral and Bone Disorders in CKD

#### 3.2.1. Osteoprotegerin (OPG) 

 OPG is considered a member of the TNF receptor family, considered to be correlated with the vascular dysfunction and further with cardiovascular disease, the common problem encountered in patients with CKD. Yilmaz et al. were among the first ones to mention the potential role of OPG in CKD patients stratification for cardiovascular risk, along with eGFR and FGF-23, in a CKD group not undergoing dialysis [41].

In this regard, according to another study, OPG increased expression could be linked to medial calcifications in aorta and renal arteries; thus its expression is recognized as a protective mechanism against vascular calcifications [6]. Thereby, the RANK/RANKL/OPG signaling pathway was found to be closely related to atherosclerosis progression [42].

**Figure 3:**
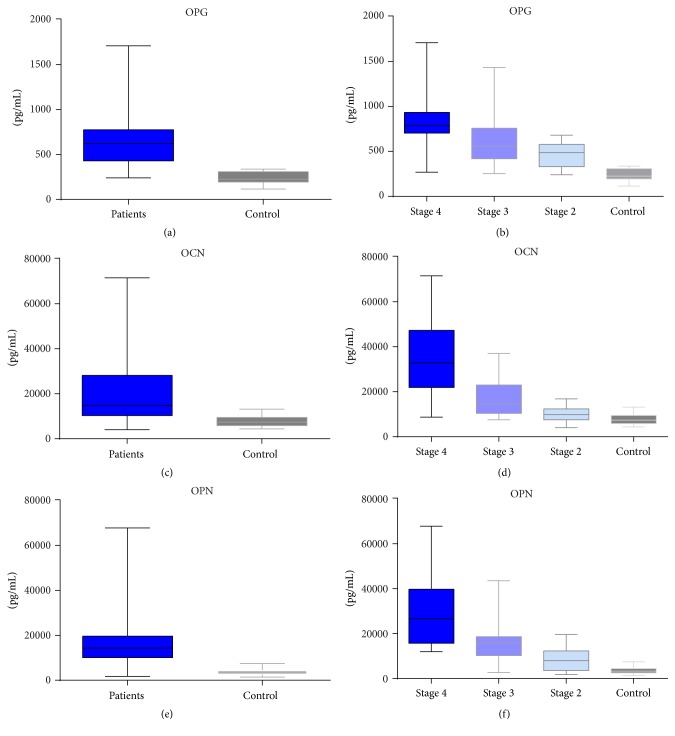
Serum level of OPG, OCN, and OPN in CKD patients compared with control, by xMAP array.

Our data suggest a statistically significant increased expression of OPG in CKD patients group compared with control (*p* < 0.001, Figure 3(a)). The same results were found in a study by Demir et al. [43]. The upward trend of OPG levels is also maintained with regard to the distribution on CKD stages, as follows: 3.5-fold higher in CKD stage 4, 2.5-fold higher in CKD stage 3, and 2-fold higher in CKD stage 2 (*p* < 0.001 for all stages, Figures 3(b) and 2).

Despite the fact that several studies propose a direct involvement action of IL-6 in the increasing level of expression of OPG, we can conclude, based on our data, that the level of OPG in serum correlates with the expression level of IL-6 in patients of CKD stage 2 only (*r* = 0.63). Given the relatively small number of patients included in this study, further research is necessary to fully understand the therapeutic and biomarker potential of OPG in patients with kidney disease.

Morena et al. were among the first that also mentioned that increased OPG levels were correlated with the progression of coronary artery calcification (CAC) in a CKD nondialyzed group [44].

It was observed that OPG knockout mice develop severe calcifications, thus the potential protective role of OPG against vascular calcification being proposed [45].

Furthermore, Lewis et al. concluded that OPG might be a crucial biomarker in CKD stages 3–5 patients with poor long-term prognosis, based on their results showing that OPG high levels were correlated with the progress in renal dysfunction [46].

#### 3.2.2. Osteocalcin (OCN)

Considering the osteoblastic activity of OCN, this marker might be directly involved in bone-vascular axis [47, 48] and its systemic and local effects could be potentially related to bone remodeling, vascular calcification, and energy metabolism [49].

We found that OCN circulating levels were increased 4.6-fold in CKD stage 4 (*p* < 0.01) and 2-fold (*p* < 0.01) and 1.3-fold (*p* = 0.05) in CKD stages 3 and 2, respectively, thus revealing an overexpression of OCN in CKD patients* versus* control (*p* < 0.001) (Figures 3(c), 3(d), and 2). The OCN serum levels, in association with inflammatory markers IL-6 and TNF-*α*, showed significant correlation with regard to CKD stage 4 only (*r* = 0.67 and *r* = 0.69) (Tables 1, 2, and 3).

Other studies observed that OCN was inversely correlated to age and IL-6, in CKD hemodialysis patients [50].

Since the controversy still exists, further research and large clinical trials are needed to clearly explain the connections between the immune system and bone-vascular axis.

#### 3.2.3. Osteopontin (OPN)

In CKD patients, starting with early stages, Barreto et al. have noticed increased OPN levels compared with control and have also related a positive correlation of OPN with the inflammatory markers [4, 51].

In a univariate linear regression assessment, OPN was found to be directly correlated with inflammation markers like IL-6, C-reactive protein (CRP), and intact parathyroid hormone (iPTH), concluding that OPN could play an important role in the pathway where inflammation enhanced CKD poor prognosis [51].

Lorenzen et al. also found a possible link between OPN and inflammation markers (IL-6, CRP) in hypertensive patients [52].

Our results were in agreement with the above-mentioned studies and revealed significant differences between the control group and patients with CKD (*p* < 0.001), being increased more than 2-fold in CKD stage 2 (*p* = 0.01), rising at 4-fold in CKD stage 3 (*p* < 0.001) and 7-fold in CKD stage 4 (*p* < 0.0001) (Figures 3(e), 3(f), and 2).

The threshold values for the analyses were established in accordance with the mean values of the studied groups. A negative correlation was observed between OPN and Fetuin-A serum levels of CKD stage 4 patients (*r* = −0.67), and a positive correlation was found between OPN and IL-6 (*r* = 0.59) and TNF-*α* (*r* = 0.72). In CKD stage 3, a statistical correlation between OPN and OPG was observed (*r* = 0.53). A statistical correlation was also found with IL-6 (*r* = 0.52) and TNF-*α* (*r* = 0.51) in CKD stage 2 (Tables 1, 2, and 3).

#### 3.2.4. Fibroblast Growth Factor 23 (FGF-23)

FGF-23 is a phosphaturic hormone with elevated levels in early CKD stages, before mineral and bone disorders become obvious [53], and might be associated with endothelial dysfunction [54] and greater risk of congestive heart failure (CHF) and atherosclerotic events in patients with CKD stages 2–4 [55].

Nonetheless, in another study, FGF23 appears not to be an early marker of CKD, in elderly patients (age over 65) [56].

It is generally considered that CKD plays the most important role in increasing FGF-23 levels; in this view, FGF-23 high levels appeared to be independently linked to CKD prognosis [57, 58], although the mechanisms are poorly understood [59].

Desjardins et al. suggest that plasma FGF-23 could be considered an independent biomarker of vascular calcification in patients with CKD, starting from early stages [60].

**Figure 4:**
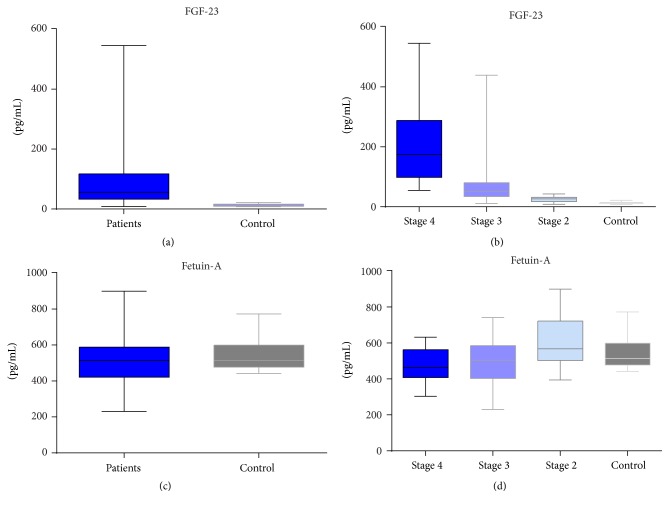
Serum level of FGF-23 and Fetuin-A in CKD patients compared with control, assessed by xMAP array.

We have found that FGF-23 levels were significantly enhanced in CKD patients (*p* < 0.001) (Figure 4(a)). Serum levels of FGF-23 showed a gradual increase, reaching the highest levels in patients with CKD stage 4 (*p* < 0.0001), being 16-fold higher than in the control group. According to previously analyzed biomarkers, serum FGF-23 levels still showed a significant increase of 6-fold (*p* < 0.001) in CKD stage 3 and 2-fold (*p* < 0.001) for CKD stage 2 (Figures 4(b) and 2). Although FGF-23 has been identified to be significantly overexpressed in CKD stages 2–4, there were no statistical correlations with the other multiplexed analyzed biomarkers (according to Pearson correlations).

#### 3.2.5. Fetuin-A

Among the multiple players involved in vascular calcification pathogenesis, Fetuin-A is considered to be an inhibitory molecule; thus CKD patients are assumed to experience a Fetuin-A deficiency, which might be considered a common feature of this disease [61].

We have noticed that the level of Fetuin-A in CKD patients was decreased compared with control (*p* = 0.15). The highest decrease was found in CKD stage 4, being of 0.85-fold (*p* = 0.02), followed by stage 3 with 0.91-fold (*p* = 0.05) and stage 2 with 1.13-fold (*p* = 0.13) (*t*-test) (Figures 4(c), 4(d), and 2). Fetuin-A presented a negative correlation with TNF-*α* (*r* = −0.61) and OPN (*r* = −0.67) in CKD stage 4, while in CKD stage 2 Fetuin-A was negatively correlated with IL-6 (*r* = −0.5) and OPG (*r* = −0.6) (Tables 1, 2, and 3).

Smith et al. also reported an association between Fetuin-A decreased levels and inflammatory markers, also with procalcific cytokine, explaining the potential involvement of this biomarker in coronary calcification and aortic stiffness [62].

### 3.3. Correlations between CKD Markers and Inflammatory Status

We have observed a strong correlation between IL-6 and eGFR (*χ* ^2^ = 16.8; *P* < 0.01), TNF-*α* (*χ* ^2^ = 7.9; *P* < 0.005), OPN (*χ* ^2^ = 5.4; *P* < 0.02), OPG (*χ* ^2^ = 8.28; *P* = 0.04), and FGF-23 (*χ* ^2^ = 5; *P* = 0.02). TNF-*α* was correlated with FGF-23 (*χ* ^2^ = 7.4; *P* = 0.006) and Fetuin-A (*χ* ^2^ = 5.9; *P* = 0.001). Strong correlations were also found between eGFR and OCN (*χ* ^2^ = 6.2; *P* = 0.01) and FGF-23 (*χ* ^2^ = 19.9; *P* < 0.001); also OCN correlated with OPN (*χ* ^2^ = 5.3; *P* = 0.02) and FGF-23 (*χ* ^2^ = 6.9; *P* = 0.008) in all CKD groups. The above-mentioned correlations, chi-square test (*χ* ^2^; *P*), between analyzed inflammatory mediators and mineral/bone disorders markers, alongside with eGFR, are shown in Table 4.

According to our results, we conclude that a crosstalk between bone, vasculature, and renal function exists in CKD, representing a major risk factor for cardiovascular morbidity and mortality.

In CKD early stage 2, an increased expression was observed for 6 out of the 7 analyzed biomarkers. From our data, circulating levels of IL-6, TNF-*α*, OPG, OCN, OPN, and FGF-23 were statistically increased (*P* < 0.05) in CKD stage 2, while Fetuin-A showed a slight alteration over control, but with no statistical significance (*P* = 0.13).

At a first glance, proteomic biomarkers offer the hope of improving the management of patients with CKD starting with early stages, yet more studies are needed to establish the diagnostic and prognostic value of these biomarkers.

## 4. Conclusions

The present study highlights the potential clinical utility of a multiplexed biomarker panel in CKD. Out of all analyzed candidate biomarkers, a panel which includes mediators of inflammation (IL-6, TNF-*α*) and mineral and bone disorder biomarkers (OPG, OPN, OCN, FGF-23, and Fetuin-A) was found to be more relevant than a single biomarker to detect patients in early CKD stages. We have noticed a positive correlation between the biomarkers panel of IL-6, OCN, and FGF-23 and renal failure progression (eGFR) in all CKD groups. The association between inflammatory cytokines and bone disorders markers, OPN, OPG, and FGF-23, reflects the severity of the vascular changes in CKD and predicts the disease progression. Proteomic xMAP analyses shed light on clinical evaluation for CKD staging and prognosis. Thus, new evidence has emerged within the relationship between bone and vascular pathology, especially in CKD patients, encouraging further investigations in the area.

## Figures and Tables

**Table 1:** Correlation between inflammatory cytokines and CKD biomarkers in stage 4 (Pearson correlation).

	IL-6	TNF-*α*	OPG	OCN	OPN	FGF-23	Fetuin-A
IL-6	1						
TNF-*α*	0.64	1					
OPG	−0.01	0.28	1				
OCN	0.67	0.69	0.21	1			
OPN	0.59	0.72	0.04	0.35	1		
FGF-23	0.24	−0.21	−0.36	0.06	0.07	1	
Fetuin-A	−0.37	−0.62	−0.31	−0.39	−0.67	0.23	1

**Table 2:** Correlation between inflammatory cytokines and CKD biomarkers in stage 3 (Pearson correlation).

	IL-6	TNF-*α*	OPG	OCN	OPN	FGF-23	Fetuin-A
IL-6	1						
TNF-*α*	0.147	1					
OPG	0.144	0.132	1				
OCN	0.240	−0.079	0.063	1			
OPN	0.173	0.106	0.53	0.083	1		
FGF-23	0.152	0.072	0.159	0.134	0.23	1	
Fetuin-A	0.196	−0.03	−0.048	0.072	0.015	−0.0006	1

**Table 3:** Correlations between inflammatory cytokines and CKD biomarkers in stage 2 (Pearson correlation).

	IL-6	TNF-*α*	OPG	OCN	OPN	FGF-23	Fetuin-A
IL-6	1						
TNF-*α*	0.583	1					
OPG	0.638	0.375	1				
OCN	0.003	0.294	0.054	1			
OPN	0.525	0.511	0.011	0.286	1		
FGF-23	−0.125	0.136	−0.123	−0.2	0.334	1	
Fetuin-A	−0.503	−0.361	−0.655	−0.275	0.158	0.28	1

**Table 4:** Correlations between inflammatory cytokines, bone and mineral disorder biomarkers, and eGFR in patients with CKD stages 2–4, not undergoing dialysis.

	IL-6			TNF-*α*			OPN			OPG			OCN			FGF-23			Fetuin-A		
	<6	≥6		<5	≥5		<6000	≥6000		<400	≥400		<12000	≥12000		<35	≥35		<400	≥400	
Gender																					
M	31	31	*P* = 0.16 *χ* ^2^ = 1.9	39	23	*P* = 0.29 *χ* ^2^ = 1.1	13	49	*P* = 0.65 *χ* ^2^ = 0.2	10	52	*P* = 0.17 *χ* ^2^ = 1.85	21	41	*P* = 0.75 *χ* ^2^ = 0.1	20	42	*P* = 0.29 *χ* ^2^ = 1.09	13	49	*P* = 0.65 *χ* ^2^ = 0.2
F	8	16		18	6		4	20		7	17		9	15		5	19		4	20	
Age																					
<60	10	12	*P* = 0.99 *χ* ^2^ = 0.0001	12	10	*P* = 0.18 *χ* ^2^ = 1.8	7	15	*P* = 0.09 *χ* ^2^ = 2.7	7	15	*P* = 0.09 *χ* ^2^ = 2.7	9	13	*P* = 0.5 *χ* ^2^ = 0.5	4	18	*P* = 0.19 *χ* ^2^ = 1.7	7	15	*P* = 0.09 *χ* ^2^ = 2.7
≥60	29	35		45	19		10	54		10	54		21	43		21	43		10	54	
eGFR																					
<60	27	47	*P* < 0.001 *χ* ^2^ = 16.8	46	28	*P* = 0.04 *χ* ^2^ = 4.02	12	62	*P* = 0.04 *χ* ^2^ = 4.2	14	60	*P* = 0.6 *χ* ^2^ = 0.2	22	52	*P* = 0.01 *χ* ^2^ = 6.2	15	59	*P* < 0.001 *χ* ^2^ = 19.9	17	64	*P* = 0.07 *χ* ^2^ = 3.08
≥60	12	0		11	1		5	7		3	9		8	4		10	2		0	12	
IL-6																					
<6				32	7	*P* < 0.005 *χ* ^2^ = 7.9	12	27	*P* < 0.02 *χ* ^2^ = 5.4	13	26	*P* = 0.004 *χ* ^2^ = 8.28	18	21	*P* = 0.04 *χ* ^2^ = 4	16	23	*P* = 0.02 *χ* ^2^ = 5	7	32	*P* = 0.7 *χ* ^2^ = 0.2
≥6				25	22		5	42		4	43		12	35		9	38		10	37	
TNF-*α*																					
<5							19	38	*P* = 0.03 *χ* ^2^ = 4.6	15	42	*P* = 0.03 *χ* ^2^ = 4.6	22	35	*P* = 0.3 *χ* ^2^ = 1.02	22	35	*P* = 0.006 *χ* ^2^ = 7.4	7	50	*P* = 0.01 *χ* ^2^ = 5.9
≥5							3	26		2	27		8	21		3	26		10	19	
OPN																					
<6000										6	11	*P* = 0.07 *χ* ^2^ = 3.2	10	7	*P* = 0.02 *χ* ^2^ = 5.3	6	11	*P* = 0.52 *χ* ^2^ = 0.39	3	14	*P* = 0.8 *χ* ^2^ = 0.06
≥6000										11	58		20	49		19	50		14	55	
OPG																					
<400													9	8	*P* = 0.08 *χ* ^2^ = 3.04	4	13	*P* = 0.57 *χ* ^2^ = 0.3	4	13	*P* = 0.66 *χ* ^2^ = 0.2
≥400													21	48		21	48		13	56	
OCN																					
<12000																14	16	*P* = 0.008 *χ* ^2^ = 6.9	6	24	*P* = 0.96 *χ* ^2^ = 0.001
≥12000																11	45		11	45	
FGF-23																					
<35																			2	23	*P* = 0.07 *χ* ^2^ = 3.07
≥35																			15	46	
Fetuin-A																					
<400																					
≥400																					
